# Integrated Multiomics Analysis Identifies a Novel Biomarker Associated with Prognosis in Intracerebral Hemorrhage

**DOI:** 10.1155/2021/2510847

**Published:** 2021-12-14

**Authors:** Yiqing Shen, Wensong Yang, Xin Xiong, Xinhui Li, Zhongsong Xiao, Jialun Yu, Fangyu Liu, Siwen Gui, Xiongfei Xie, Fajin Lv, Libo Zhao, Liangbo Hu, Anatol Manaenko, Peng Xie, Qi Li

**Affiliations:** ^1^Department of Neurology, The First Affiliated Hospital of Chongqing Medical University, Chongqing 400016, China; ^2^NHC Key Laboratory of Diagnosis and Treatment on Brain Functional Diseases, The First Affiliated Hospital of Chongqing Medical University, Chongqing 400016, China; ^3^Department of Neurology, Chongqing Hospital of Traditional Chinese Medicine, Chongqing 400011, China; ^4^Department of Radiology, Yongchuan Hospital of Chongqing Medical University, Chongqing 402160, China; ^5^Department of Radiology, The First Affiliated Hospital of Chongqing Medical University, Chongqing 400016, China; ^6^Department of Neurology, Yongchuan Hospital of Chongqing Medical University, Chongqing 402160, China; ^7^Chongqing Key Laboratory of Cerebrovascular Disease Research, Yongchuan Hospital of Chongqing Medical University, Chongqing 402160, China

## Abstract

Existing treatments for intracerebral hemorrhage (ICH) are unable to satisfactorily prevent development of secondary brain injury after ICH and multiple pathological mechanisms are involved in the development of the injury. In this study, we aimed to identify novel genes and proteins and integrated their molecular alternations to reveal key network modules involved in ICH pathology. A total of 30 C57BL/6 male mice were used for this study. The collagenase model of ICH was employed, 3 days after ICH animals were tested neurological. After it, animals were euthanized and perihematomal brain tissues were collected for transcriptome and TMT labeling-based quantitative proteome analyses. Protein-protein interaction (PPI) network, Gene Set Enrichment Analysis (GSEA), and regularized Canonical Correlation Analysis (rCCA) were performed to integrated multiomics data. For validation of hub genes and proteins, qRT-PCR and Western blot were carried out. The candidate biomarkers were further measured by ELISA in the plasma of ICH patients and the controls. A total of 2218 differentially expressed genes (DEGs) and 353 differentially expressed proteins (DEPs) between the ICH model group and control group were identified. GSEA revealed that immune-related gene sets were prominently upregulated and significantly enriched in pathways of inflammasome complex, negative regulation of interleukin-12 production, and pyroptosis during the ICH process. The rCCA network presented two highly connective clusters which were involved in the sphingolipid catabolic process and inflammatory response. Among ten hub genes screened out by integrative analysis, significantly upregulated Itgb2, Serpina3n, and Ctss were validated in the ICH group by qRT-PCR and Western blot. Plasma levels of human SERPINA3 (homologue of murine Serpina3n) were elevated in ICH patients compared with the healthy controls (SERPINA3: 13.3 ng/mL vs. 11.2 ng/mL, *p* = 0.015). Within the ICH group, higher plasma SERPINA3 levels with a predictive threshold of 14.31 ng/mL (sensitivity = 64.3%; specificity = 80.8%; AUC = 0.742, 95% CI: 0.567-0.916) were highly associated with poor outcome (mRS scores 4-6). Taken together, the results of our study exhibited molecular changes related to ICH-induced brain injury by multidimensional analysis and effectively identified three biomarker candidates in a mouse ICH model, as well as pointed out that Serpina3n/SERPINA3 was a potential biomarker associated with poor functional outcome in ICH patients.

## 1. Introduction

Intracerebral hemorrhage (ICH), which accounts for 15-20% of all strokes, is the most devastating stroke subtype, contributing to high mortality and disability in the world [1–3]. However, no effective pharmaceutical or surgical treatments, which can treat ICH-induced brain injury, are available, and as such, novel exploration of ICH-related biomarkers and therapeutic targets is urgently needed [4].

Secondary injury develops soon after ICH onset and is mostly driven by the inflammatory response, edema, oxidative stress, and excitotoxicity [5, 6]. Preclinical studies have demonstrated that cascades of inflammatory processes occur around the hematoma [7–10]. Almost simultaneously, mitochondrial dysfunctions, oxidative stress, and neurotoxicity mediated injury could result in the blood-brain barrier (BBB) impairment and aggregate the formation of perihematomal edema [11–14]. Generally speaking, secondary brain injury following ICH is not caused by a single factor, but a cascade of amplified reactions caused by multiple pathological factors working in concert. In this regard, deepened insight into the underlying mechanism of ICH injury is required to aid in identifying molecular indicators of ICH and provide potential intervention targets for clinical treatment.

Progress in omics-based approach with high-throughput data is emerging as an available approach to enhance the understanding of the pathophysiology of ICH. Transcriptomics technologies, which focus on both coding and noncoding sequences [5], have been applied to brain tissue and peripheral blood. Previous studies have identified that several transcript panels are related to ICH diagnosis and prognosis [15–17]. Meanwhile, proteomics approaches have made it an ideal strategy for molecular mechanism studies in ICH [18–21]. However, given the complexity and variability of pathophysiological processes involved in brain injury after ICH, such independent analysis from each level of omics may overlook the crosstalk between different molecular entities and could miss biologically relevant information [22]. In this context, integrated analysis of multiomics (intergromics) has emerged as a novel approach to facilitate interpretation of the multidimensional data and provide a global-wide insight into the pathological changes of ICH.

In the present study, we firstly explored the main transcriptomics and proteomics changes that occur in the brains of ICH mice. Then, we combined experimental data of two omics simultaneously by using integrative analysis and further identified the key significant expression molecules in the acute phase of ICH. Finally, we assessed the association between the plasma level of highlighted candidate molecule and clinical characteristics of ICH patients.

## 2. Materials and Methods

### 2.1. Animals

30 C57BL/6 male mice (8-12 weeks old, 20-25 g) were purchased from the Laboratory Animal Center of Chongqing Medical University. All the animals were individually housed under the standard conditions (temperature of 21-24°C, humidity of 52°, 12 h light-dark cycle). All animal experiments were approved by the Ethical Committee of Chongqing Medical University (ECCMU, Chongqing, China) and conducted in compliance with the National Institutes of Health Guidelines for animal research. Utmost efforts were taken to reduce the number and minimize suffering of animals.

### 2.2. Experimental ICH Model

ICH was induced in mice by collagenase injection under inhalation anesthesia with isoflurane as previously reported [23]. The mice were fixed in a stereotactic frame (RWD, Life Science, China) in a prone position. The scalp was incised along the midline, and a burr hole was drilled. The mice were injected in the right striatum (coordinates: 0.6 mm anterior, 3.7 mm ventral, and 2.3 mm lateral to the bregma) with type IV-S clostridial collagenase (Sigma, St. Louis, MO, USA; 0.075 U in 0.4 *μ*L sterile 0.9%NaCl) by using a 26-gauge Hamilton syringe needle. The needle was left for 10 min and then slowly withdrawn after it. Control surgeries were performed using the same surgical procedures without injection of type IV collagenase. Exclusion criterion was death during or after surgery.

### 2.3. Behavioral Assessment

Neurological function evaluation was performed using the modified Garcia test and beam walking test at 3 days after ICH, as previously described [24]. The modified Garcia test consisted of 6 subtests including vibrissae touch, trunk touch, spontaneous activity, spontaneous movement of the four limbs, forelimbs outstretching, and climbing capacity. A maximal score of 18 was given to animals without apparent neurological dysfunctions. The beam walking score was determined by observing the walking distance of mice on a wooden beam within 1 minute: five points, reaching the opposite platform or walking ≥ 45 cm within 30 s; four points, reaching the opposite platform or walking ≥ 45 cm within 60 s; three points, walking ≥ 22.5 cm within 60 s; two points, walking < 22.5 cm within 60 s; one point, no walking but no falling over 30 s (any posture); and zero points, falling within 30 s [25]. The higher score, the better neurological function. All tests were performed by a blinded investigator.

### 2.4. Sample Collection and Preparation

Mice were deeply anesthetized with pentobarbital sodium. Mouse brain samples were quickly removed and sliced into 1 mm coronal sections. The perihematomal brain tissues were obtained and then stored at -80°C for subsequent experiments, including microarray analysis, liquid chromatography-mass spectrometry/mass spectrometry (LC-MS/MS) analysis, quantitative RT-PCR (qRT-PCR), and Western blot.

### 2.5. Hematoxylin-Eosin (HE) Staining

Mice were deeply anesthetized and then transcardially perfused with 40 mL precold phosphate buffer saline (PBS). The brains were removed, fixed with 4% paraformaldehyde and embedded in paraffin. Then, 3 *μ*m thick brain tissue sections were prepared. After the deparaffinization, the brain tissue sections were stained with hematoxylin/eosin according to the manufacturer's instructions. Sections were viewed using a light microscope (Eclipse Ci-L, Nikon, Japan).

### 2.6. Transcriptomics Study

Total RNA extraction was performed from brain tissues (*n* = 5, each group). NanoPhotometer® spectrophotometer (IMPLEN, CA, USA) and Bioanalyzer 2100 system (Agilent Technologies, CA, USA) were used to evaluat the purity and integrity of RNA. NEBNext® Ultra™ RNA Library Prep Kit for illumina® (NEB, USA) was used to generate a sequencing library according to the manufacturer's recommendations. The TruSeq PE Cluster Kit v3-cBot-HS was used to generate clusters, and the library preparation was sequenced on the illumina Novaseq platform. After a high-quality process and clean of the raw data, Hisat2 v2.0.5 was used to build the index of the reference genome and align paired-end clean reads to the reference genome. Then, we calculate the FPKM of each gene based on the length of the gene and the count of reads mapped to that gene. DESeq2 (1.16.1) and edgeR (3.18.1) were used to analyze the differential expression of the two groups. Differentially expressed genes (DEGs) were defined as the ones with log_2_ | fold change (FC) | ≥1.2 and *p* < 0.05.

### 2.7. Proteomics Study

#### 2.7.1. Total Protein Extraction and Protein Quality Test

The sample was ground in liquid nitrogen and lysed with lysis buffer (100 mM NH4HCO3, 8 M Urea and 0.2% SDS). After sonication, the lysate was centrifuged for 15 minutes (4°C, 12000 g), and the supernatant was transferred to a clean tube. The supernatant was reduced with 10 mM DTT and then alkylated with sufficient iodoacetamide. The sample was thoroughly mixed with prechilled acetone by vortex, and incubated at -20°C for at least 2 hours. After it, samples were centrifuged (4°C, 12000 g), and precipitates were collected. After washing twice with cold acetone, the precipitates were dissolved in buffer (0.1 M triethylammonium bicarbonate and 6 M urea).

#### 2.7.2. TMT Labeling of Peptides

The protein sample (*n* = 3, each group) was diluted with solubilization buffer and added with trypsin and TEAB buffer. The mixed sample was digested with trypsin overnight. The digested sample was mixed with formic acid and centrifuged (4°C, 12000 g) for 5 minutes at room temperature. The supernatant was slowly loaded onto the C18 desalting column, washed with washing buffer (0.1% formic acid, 3% acetonitrile) 3 times, and then eluted with elution buffer (0.1% formic acid, 70% acetonitrile). The eluate from each sample was collected and lyophilized. Subsequently, each sample was added 100 *μ*L of 0.1 M TEAB buffer and 41 *μ*L of TMT labeling reagent dissolved in acetonitrile. Finally, 8% ammonia was added to terminate the reaction. All labeled samples were mixed in equal volume, desalted, and lyophilized.

#### 2.7.3. Separation of Fractions

Mobile phases A (2% acetonitrile, adjusted pH to 10.0 using ammonium hydroxide) and B (98% acetonitrile) were used to develop a gradient elution. The lyophilized powder was dissolved in solution A and centrifuged at 12,000 g for 10 min at room temperature. The sample was fractionated using a C18 column (Waters BEH C18 4.6 × 250 mm, 5 *μ*m) on a Rigol L3000 HPLC system; the column oven was set as 50°C.

#### 2.7.4. LC-MS/MS Analysis

For transition library construction, shotgun proteomics analyses were performed using an EASY-nLCTM 1200 UHPLC system (Thermo Fisher) coupled with a Q Exactive HF-X mass spectrometer (Thermo Fisher) operating in the data-dependent acquisition (DDA) mode.

#### 2.7.5. Protein Identification and Quantification

In order to improve the quality of the analysis results, the search results were further filtered through the software PD 2.2. The identified protein contains at least 1 unique peptide. The identified PSMs and protein were retained and performed with false discovery rate (FDR) ≤ 1%. The significance of protein quantitation results was statistically analyzed by a *t*-test. Differentially expressed proteins (DEPs) were defined as the ones with FC ≥ 1.2 or FC ≤ 0.83 and *p* < 0.05.

### 2.8. Functional Enrichment Analysis of DEGs and DEPs

ClusterProfiler R package (version 3.14.3) was used to perform the gene ontology (GO) and Kyoto Encyclopedia of Genes and Genomes (KEGG) pathway enrichment analysis, and ggplot R package (version 3.3.3) was imported to visualize the results.

### 2.9. Gene Set Enrichment Analysis (GSEA)

GSEA is mainly used to determine whether a predefined gene set can show significant differences between two biological states. We conducted GSEA analysis through the local version (http://www.broadinstitute.org/gsea/index.jsp) and obtained important gene sets through the following parameters: NES > 1, NOM *p* value < 0.01, and FDR *q* value < 0.25.

### 2.10. Integrative Analysis

All DEGs and DEPs screened from transcriptomics data and proteomics data, respectively, were included in the follow-up analysis. To reveal the interaction networks underlying multidimensional data, different integrated bioinformatics approaches were used through the following four steps.

Firstly, to obtain an overview of the expressive and regulated characteristics of DEGs and DEPs, the R package (version 3.6.3) was used to create a Venn diagram, and hierarchical clustering analysis was performed based on the normalized values of proteins. Then, Pearson's correlation analysis was performed to assess the correlation of expression levels between two high-throughput datasets.

Secondly, for those persistently dysregulated DEGs and DEPs, a protein-protein interaction (PPI) network with a strict combined score > 0.7 was created by the online database STRING (version 11.0) (https://string-db.org/) and was visualized by Cytoscape (version 3.8.0), a public bioinformatics analysis platform. Furthermore, CytoHubba was used to identify hub genes based on degree algorithms.

Thirdly, the mixOmics R package (version 6.3.1, http://mixomics.org) was used to perform a regularized Canonical Correlation Analysis (rCCA) to investigate the most significantly correlated module between transcriptomics and proteomics data. As reported previously [26], omics data were repeatedly shrunk and optimized according to regularize the empirical covariance matrices of variables (*X*, *Y*) and tune two parameters (*λ*1, *λ*2) using cross-validation until parameter *λ*1 = 0.05, parameter *λ*2 = 0.2, and the cross-validation score = 0.87. Then, Biological Networks Gene Ontology (BiNGO) package (version 3.0.3) was used to conduct the biological process analysis of highly relevant networks (parameter setting: significance level: 0.05, weight: 0.8). The rCCA network and PPI network were combined with integrate consideration, and hub molecules were finally screened out.

### 2.11. qRT-PCR

Total RNA was extracted from mouse brain tissues by Trizol (Invitrogen, United States). For cDNA synthesis, the PrimeScript™ RT reagent kit was performed followed the manufacturer's instructions (RR047A, TaKaRa, Shiga, Japan). qRT-PCR was carried out using the SYBR Green detection system (Roche, Germany) according to the manufacturer's instructions. *β*-Actin was used as the internal control in all samples. The values of fold change for mRNA expression levels were calculated according to formula 2^-ddCT^. The primer sequences are presented in Supplementary Table S1.

### 2.12. Western Blot

Briefly, whole-cell lysates were obtained by gently homogenizing of brain samples in RIPA lysis buffer (P0013, Beyotime, China) and centrifuging (14,000 g at 4°C for 30 min). The supernatant was collected, and the protein concentration was determined using a bicinchoninic acid (BCA) assay (Bio-Rad, Dc protein assay). Equal amounts of protein (30 *μ*g) were loaded and subjected to electrophoresis on a 10% SDS-PAGE gel. After being transferred onto a PVDF membrane (Bio-Rad), the membrane was blocked with 0.5% skimmed milk in TBST for 3 h at room temperature and was incubated with the primary antibody overnight at 4°C. Then, the membrane was incubated with corresponding secondary antibodies for 2 h at room temperature. Internal loading control *β*-actin was used. The primary antibodies were Serpina3n (1 : 500, AF4709, R&D Systems), Itgb2 (1 : 1000, 10554-1-AP, ProteinTech Group), Serping1 (1 : 1000, 12259-1-AP, ProteinTech Group), and Ctss (1 : 1000, DF8246, Affinity). *β*-Actin (1 : 10000, ab8226, Abcam) was used as the internal loading control. The blots were visualized using an ECL Kit (Millipore) and ChampChemi (Sage Creation Science, Beijing, China). The density of the bands was analyzed by Quantity One software (Bio-Rad, California, USA).

### 2.13. Human Blood Samples and Biomarker Measurement

All blood samples of ICH patients were withdrawn and collected in EDTA tubes within 24 hours from ICH onset. Afterward, blood samples and controls were centrifuged at 3000 rpm for 10 minutes. Separated plasma from the top layer was preserved at -80°C until measurements. Plasma concentrations were gauged in duplicate samples with commercial enzyme-linked immunosorbent assay (ELISA) kits (KALANG Corp).

Clinical information was further collected, including baseline demographics, clinical admission status, laboratory data, and imaging data. A routine 90-day follow-up was conducted for ICH patients. Functional outcomes of ICH patients were assessed by modified Rankin Scale (mRS) at a 90-day follow-up, as described previously [27]. mRS scores 4-6 were defined as poor outcomes.

### 2.14. Statistical Analysis

Most of the methods of bioinformatics analysis have been described above. Besides, the SPSS package (version 24.0) and R (version.3.6.3) software were used for statistical analyses. All data were presented as the mean and standard deviation (mean ± SD). Student's *t*-test or one-way ANOVA was performed for statistical evaluation. The two-side *p* value < 0.05 was considered statistically significant at a 95% confidence level.

## 3. Results

### 3.1. Histological and Functional Evaluation of Experimental ICH Model

**Figure 1:**
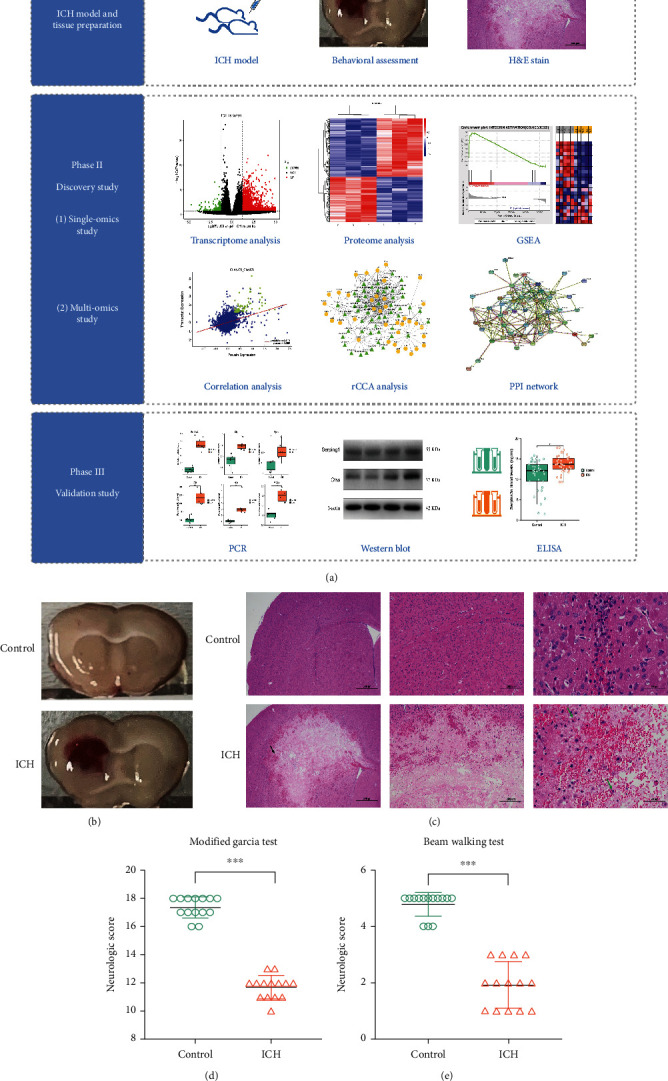
The workflow of this study and histological evaluation of the ICH mouse model. (a) Overall workflow chart of this study. (b) Representative images of brain Section (1 mm thick) in the control group and ICH group. (c) HE staining of brain tissue of control mice and ICH mice. Low-magnification (×40) images show the overall hematoma area following ICH (black arrow), high-magnification (×100, ×400) images show the significant histopathological changes (green arrows). *n* = 4 per group. (d) Statistical analysis of modified Garcia test in the ICH group and control group 3 days after operation. (e) Statistical analysis of beam walking test in the ICH group and control group 3 days after operation. *n* = 16 per group. ^∗^*p* < 0.05; ^∗∗^*p* < 0.01; ^∗∗∗^*p* < 0.001.

The overall workflow of this study is shown in Figure 1(a). Briefly, the workflow included three phases: (i) ICH modeling and tissue preparation, (ii) discovery study, and (iii) validation study. Of the 30 mice, 2 mice in the ICH group died due to excessive hematoma. The total mortality of the surgeries was 6.67% (2/30).  Figure 1(b) shows representative images of ICH and sham brains, which verify the morphological and pathological changes.  Figure 1(c) demonstrates the representative brain slices after H&E staining. Of note, macrophage infiltration (green arrows) and hemorrhages (black arrow) could be seen on the left side of the striatum in ICH mice compared with the controls. ICH induced severe neurological dysfunction, which was evaluated by modified Garcia and beam walking tests compared with the controls. (Figure 1(d)).

### 3.2. DEGs in the Perihematomal Brain following ICH

**Figure 2:**
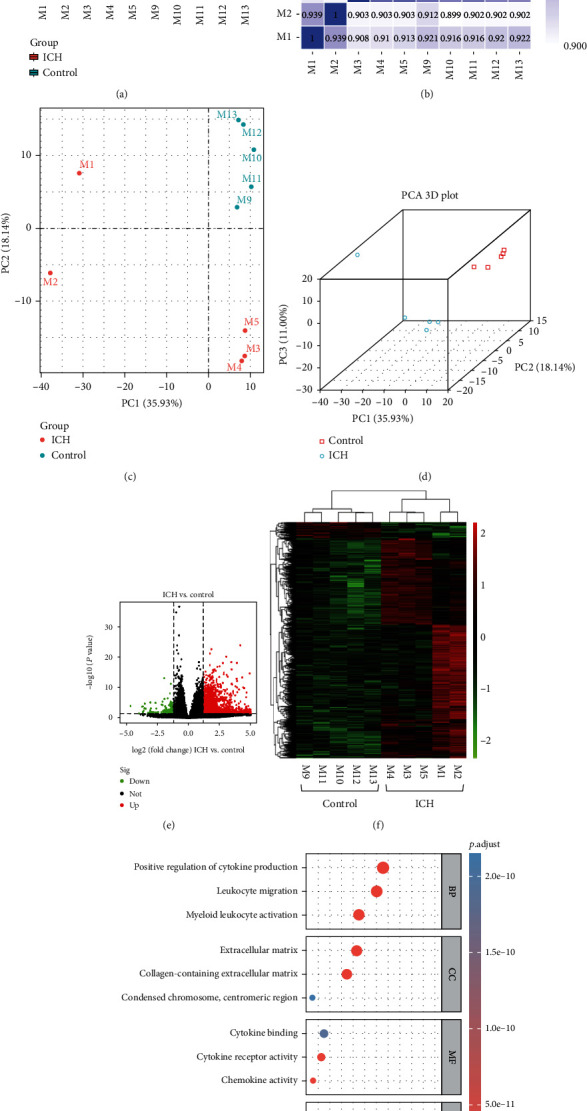
Expression and functional enrichment analysis of DEGs following ICH. (a) Box plots of gene expression distribution between the ICH and control groups. (b) Heatmap of the correlation of gene expression levels between the ICH and control groups. (c) Two-dimensional PCA analysis between ICH and control samples. (d) Three-dimensional PCA analysis between ICH and control samples. (e) Volcano plots of 2218 DEGs among transcriptomic profile between the ICH and control groups. (f) Heatmap of hierarchical clustering analysis of 2218 DEGs between the ICH and control groups. (g) Top GO and KEGG pathways enriched by DEGs between the ICH and control groups. ICH group: mice with M1-M5; control group: mice with M9-M13.

The ipsilateral hemisphere was obtained to perform transcriptomic analysis. The box plot showed the distribution of gene expression levels in the ten perihematomal brain tissues samples ICH compared to the controls (Figure 2(a)). The correlation of gene expression levels between all samples is shown in Figure 2(b). The principal component analysis (PCA) plots showed that the samples in the ICH group and control group were separated in both 2D and 3D dimensions, indicating the gene expression pattern between these two groups was different (Figures 2(c) and 2(d)). These analysis results showed the reliability of the omics data was guaranteed.

A total of 2218 genes (2065 upregulated and 153 downregulated) were identified between the ICH group and control group as DEGs (Figure 2(e)). Hierarchical clustering analysis showed that these DEGs could significantly distinguish ICH vs. sham groups (Figure 2(f)). To further identify the function of DEGs, GO enrichment analysis and KEGG pathway enrichment analysis were subjected by using clusterProfiler R package. As shown in Figure 2(g) and Table 1, DEGs were significantly enriched in 1044 GO biological process (BP) terms, which identified leukocyte migration, myeloid leukocyte activation, and positive regulation of cytokine production as the top BP terms. The DEGs were significantly enriched in 56 GO cellular component (CC) terms, which identified collagen-containing extracellular matrix, extracellular matrix and condensed chromosome, and centromeric region as the top CC terms. Furthermore, the DEGs were observed in 101 GO molecular function (MF) terms, which identified chemokine activity, cytokine receptor activity, and cytokine binding as the top MF terms. As for KEGG pathway enrichment analysis, the results demonstrated that a total of 56 pathways were significantly enriched, which were mainly enriched in cytokine-cytokine receptor interaction, viral protein interaction with cytokine and cytokine receptor, and hematopoietic cell lineage. These findings indicated that immune-related changes were deeply involved in the acute stage of ICH.

### 3.3. DEPs in the Perihematomal Brain Proteome following ICH

**Figure 3:**
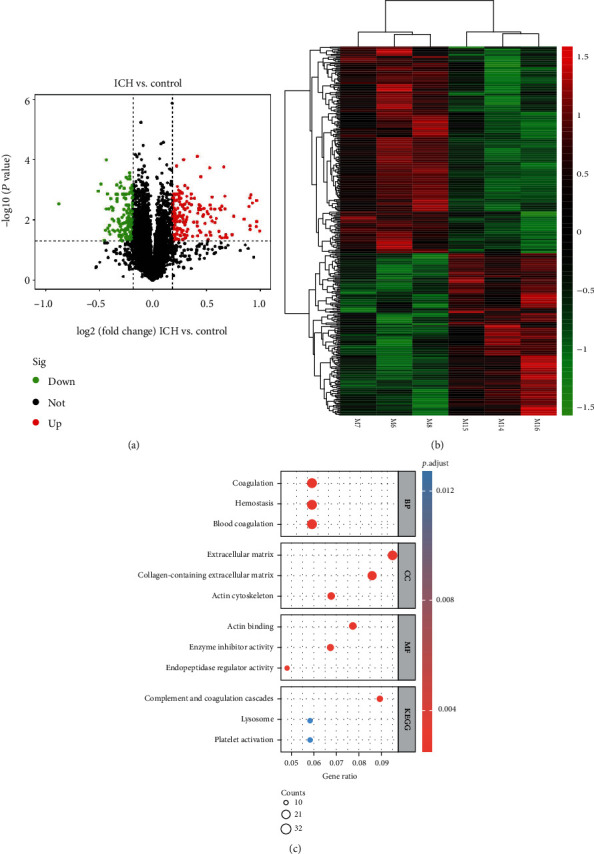
Expression and functional enrichment analysis of DEPs following ICH. (a) Volcano plots of 353 DEPs among proteomics profile between the ICH and control groups. (b) Heatmap of hierarchical clustering analysis of 353 DEPs between the ICH and control groups. (c) Top GO and KEGG pathways enriched by DEGs between the ICH and control group. ICH group: mice with M6-M8; control group: mice with M14-M16.

The TMT-labelled quantitative proteomics analysis of perihematomal brain tissues was performed. First, we matched the peptides in the database, removed duplicate proteins, and eliminated the peptides and proteins with FDR greater than 1%. Then, a total of 6012 proteins were identified in the ICH group and control group, of which 353 proteins (198 upregulated and 155 downregulated) were screened out as DEPs (Figure 3(a)). Hierarchical clustering analysis results showed that these DEPs could significantly distinguish the ICH and control groups (Figure 3(b)). As shown in Figure 3(c) and Table 2, GO enrichment analysis and KEGG pathway enrichment analysis were performed using clusterProfiler R package. DEPs were significantly enriched in 670 GO BP terms, which identified blood coagulation, hemostasis, and coagulation as the top BP terms. DEPs were also significantly enriched in 73 GO CC terms, which identified collagen-containing extracellular matrix, extracellular matrix, and actin cytoskeleton as the top CC terms. Furthermore, these DEPs were enriched in 41 GO MF terms, which played essential roles in terms of actin binding, enzyme inhibitor activity, and endopeptidase regulator activity. Next, KEGG pathway enrichment analysis showed the results that DEPs were enriched 6 KEGG pathways, such as complement and coagulation cascades and lysosome.

### 3.4. Functional Analysis through GSEA

**Figure 4:**
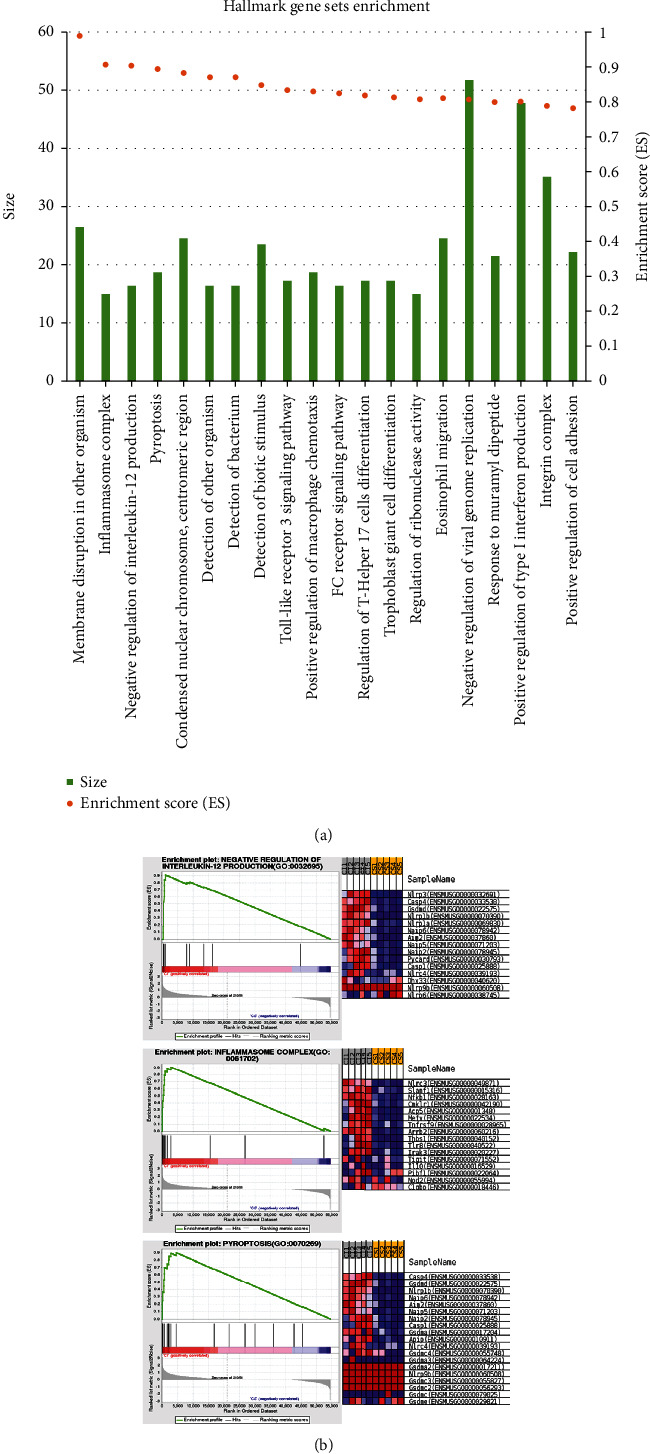
GSEA analysis of transcriptomic profile between the ICH group and control group. (a) A rank of top 20 significantly enriched gene sets with their ES. (b) A panel of immune-related gene sets represented with enplots and heatmaps of related gene expression.

To validate omics results and search for those gene sets with important biological functions, GSEA was subsequently performed based on overall genes following GO annotation. As shown in Figure 4(a), the top 20 enriched GO terms were presented ranking by enrichment scores (ES). The enrichment maps were obtained and summarized in Figure 4(b), which showed that immune-related gene sets were prominently upregulated during the ICH process compared with the controls, involving pathways including inflammasome complex, negative regulation of interleukin-12 production, and pyroptosis.

### 3.5. Integrative Multiomics Analysis following ICH

**Figure 5:**
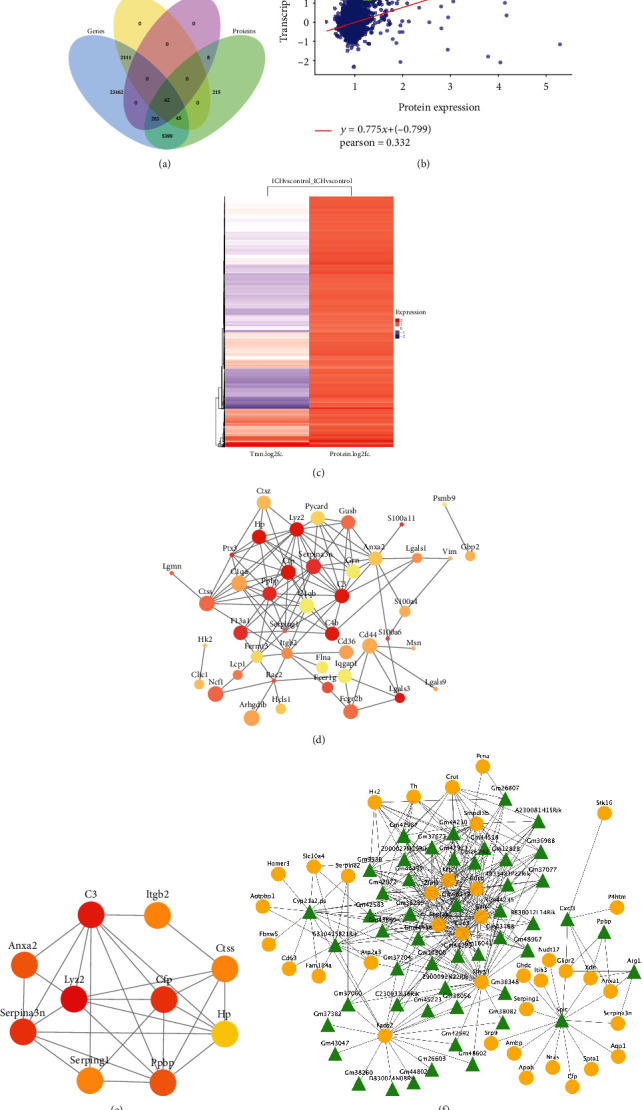
Integrative analysis of transcriptomics and proteomics datasets. (a) Venn diagram of relationship between genes and proteins. (b) Pearson's correlation analysis of the expression of comolecules. The green spots represent differentially expressed proteins in the ICH group when compared with the controls, and the blue spots represent not differentially expressed proteins in the ICH group when compared with the controls. (c) Heatmap of the expression of comolecules between transcriptomics and proteomics datasets. (d) PPI network of 62 comolecules. The node size indicates the combined score, and the nodes color indicate the log_2_|FC| (red: high log_2_|FC| value, yellow: low log_2_|FC| value). (e) Identification of hub molecules derived from PPI network of 62 comolecules. (f) rCCA network between DEG and DEP datasets. The green triangle indicates mRNAs, and the yellow circle indicates proteins.

We performed integrative analysis to reveal the post transcriptomics regulation of gene expression and explore ICH-related hub molecules. We explored the corresponding relationship between the mRNA information obtained by the transcriptome and the protein information identified by the proteome. The result showed that a total of 5789 comolecules simultaneously changed in both transcriptomic and proteomics datasets. Among these comolecules, 62 molecules were identified as simultaneously differentially expressed in both two omics data, which might serve as molecular markers of ICH (Figure 5(a)). Further, the transcriptome and proteome expression correlation analysis revealed that Pearson's correlation coefficient of comolecules was 0.332 (*p* = 0.011) (Figures 5(b) and 5(c)).

For the 62 comolecules, we established a PPI network with 44 nodes and 109 interactions (Figure 5(d)). To further identify the key ICH-related molecules of PPI network, we screened the top 10 molecules ranked by degree via the CytoHubba plug-in, namely, Lyz2, C3, Serpina3n, Cfp, Ppbp, Hp, Serping1, Ctss, Itgb2, and Anxa2 (Figure 5(e)). Meanwhile, by constructing a gene-protein connection from DEGs and DEPs, we found two highly correlated clusters in the rCCA network, of which cluster 1 contained 67 nodes with 275 interactions and cluster 2 contained 21 nodes with 27 interactions (Figure 5(f)). To further identify the fundamental function of the molecular in two clusters, GO-BP analysis was performed using BiNGO plug-in. In cluster 1, relative molecules were greatly involved in the sphingolipid catabolic processes, membrane lipid catabolic process, cerebellar cortex morphogenesis, neurotransmitter metabolic process, and cerebellum morphogenesis. In cluster 2, the top 5 GO-BP terms were mainly response to wounding, response to stress, inflammatory response, acute inflammatory response, and response to stimulus, respectively (Table 3), which indicated their importance in the process of intracerebral hemorrhage. Then, we matched 10 hub molecules with genes/protein in the rCCA network. In this regard, three candidates (Serpina3n, Serping1, and Cfp) were screened out, which presented a tight interaction with other molecules.

### 3.6. Validation of Hub DEGs/DEPs following Integrative Analysis

**Figure 6:**
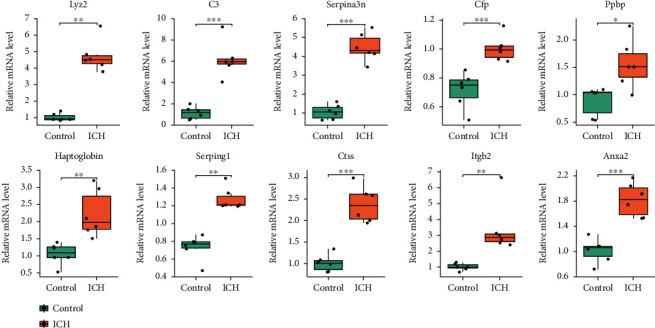
Validation of ten hub genes by qRT-qPCR. qRT-qPCR established that expression of following genes were upregulated in ICH *vs.* control brains of mice: Lyz2, C3, Serpina3n, Cfp, Ppbp, Haptoglobin, Serping1, Ctss, Itgb2, and Anxa2 in the ICH and control groups. *n* = 6 per group. ^∗^*p* < 0.05; ^∗∗^*p* < 0.01; ^∗∗∗^*p* < 0.001.

A total of 10 hub genes were chosen to further validate their expression in mRNA levels by qRT-qPCR (Figure 6). The results showed significant difference between the ICH and control groups in the expression of the following genes: Lyz2 (*p* = 0.002), C3 (*p* < 0.001), Serpina3n (*p* < 0.001), Cfp (*p* = 0.001), Ppbp (*p* = 0.030), Haptoglobin (*p* = 0.004), Serping1 (*p* = 0.005), Ctss (*p* < 0.001), Itgb2 (*p* = 0.002), and Anxa2 (*p* < 0.001), which was consistent with the results of gene expression profiling in transcriptomics. Then, we performed Spearman's correlation analysis between the mRNA expression levels of ten hub genes and the scores of two behavioral tests (Figure S1). The results showed that Haptoglobin mRNA expressive level had a very strong correlation with mGarcia scores (*r* = −0.83, *p* = 0.001). The mRNA expression levels of Lyz2 (*r* = −0.74, *p* = 0.06), C3 (*r* = −0.60, *p* = 0.039), Serpina3n (*r* = −0.7, *p* = 0.012), Cfp (*r* = −0.73, *p* = 0.007), Serping1 (*r* = −0.71, *p* = 0.01), Ctss (*r* = −0.69, *p* = 0.012), and Itgb2 (*r* = −0.73, *p* = 0.007) had strong correlations with mGarcia scores, while the Ppbp mRNA expression had no significant correlation with Garcia scores (*r* = −0.56, *p* = 0.06). Besides, the results also showed that Serpina3n mRNA expression had very strong correlation with beam walking scores (*r* = −0.86, *p* < 0.001), and the rest nine hub genes mRNA expression had strong correlations with beam walking scores.

**Figure 7:**
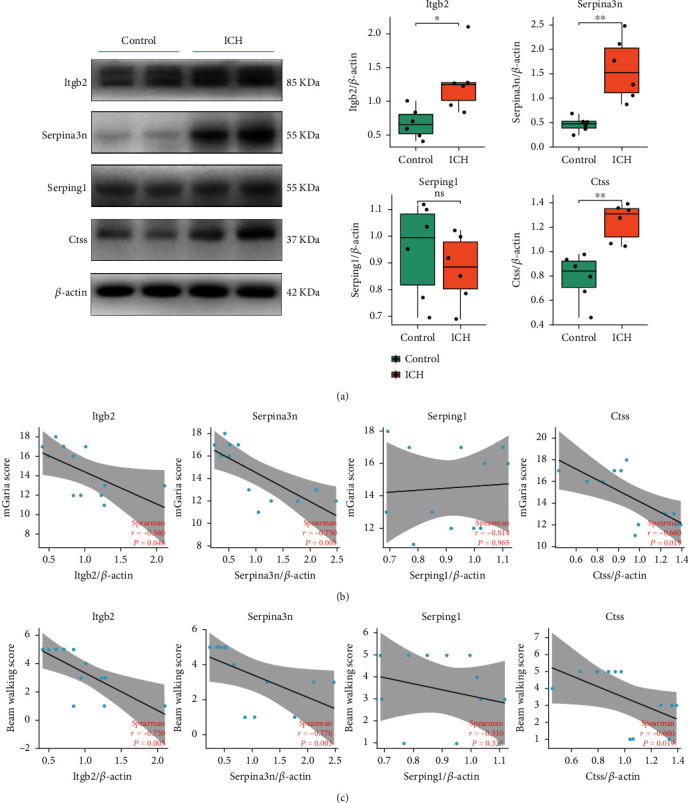
Validation of four candidates by Western blot and the correlation analysis between four candidates and behavior tests. (a) Representative Western blot bands and quantitative analysis of Itgb2, Serpina3n, Serping1, and Ctss protein expression. (b) Spearman's correlation analysis results showing the correlation between the protein expression levels of candidates and the scores of mGarcia test. (c) Spearman's correlation analysis results showing the correlation between the protein expression levels of candidates and the scores of beam walking test. *n* = 6 per group. ^∗^*p* < 0.05; ^∗∗^*p* < 0.01; ^∗∗∗^*p* < 0.001.

Given the high correlation between mRNA expression and behavioral assessments and no relevant report in ICH field, we selected four novel candidates (Serpina3n, Serping1, Ctss, and Itgb2) from ten hub molecules and explore their protein expression. Results showed significant upregulation of Serpina3n (*p* = 0.006), Itgb2 (*p* = 0.014), and Ctss (*p* = 0.001) in brain tissues of ICH compared to the control (Figure 7(a)). The results of correlation analysis showed that protein expression of Serpina3n and Ctss showed a strong correlation with mGarcia scores. Additionally, Itgb2 protein expression exhibited a moderate correlation with results of the mGarcia test. Serpina3n, Itgb2, and Ctss protein expressions were all strongly correlated with beam walking scores. However, neither mGarcia scores nor beam walking scores had a significant correlation with Serping1 protein expression (Figures 7(b) and 7(c)).

### 3.7. Exploration of the Diagnostic and Prognostic Value of Serpina3n as a Biomarker

**Figure 8:**
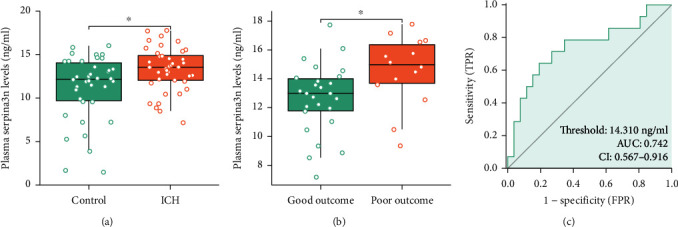
Predictive efficacy of SERPINA3N in diagnosis and prognosis in ICH patients. (a) Dot plots with plasma SERPINA3 levels in ICH patients and the controls. (b) Dot plots with plasma SERPINA3 levels between patients with good outcome and patients with poor outcome. (c) ROC curves of plasma SERPINA3 levels for prediction of 90-day prognosis of ICH patients. ^∗^*p* < 0.05; ^∗∗^*p* < 0.01; ^∗∗∗^*p* < 0.001.

In the current study, we enrolled 40 ICH patients and 38 healthy controls. The demographic and clinical information of those two groups was collected and is presented in Supplementary Table S2. The control group matches ICH patients in terms of age and gender. Of interest, plasma concentrations of SERPINA3 were significantly increased in ICH patients compared with the controls (13.3 ng/mL vs. 11.2 ng/mL, *p* = 0.015) (Figure 8(a) and Supplementary Table S2).

Of the 40 patients, we noticed that there were no significant differences in demographic indicators. However, patients with poor outcomes had higher admission SBP (*p* = 0.081), lower admission Glasgow Coma Scale (GCS) score (*p* < 0.001), higher admission National Institute of Health Stroke Scale (NIHSS) score (*p* < 0.001), larger baseline hematoma volume (*p* = 0.029), and more frequent intraventricular hemorrhage (IVH) on initial computed tomography (CT) (*p* = 0.002, Supplementary Table S3). Strikingly, the patients with a poor outcome exhibited higher plasma Serpina3n levels than patients with a good outcome (Supplementary Table S3, Figure 8(b)). According to receiver operating characteristic (ROC) curve (Figure 8(c)), choosing plasma Serpina3n levels ≥ 14.310 ng/mL as a threshold value exhibited the best diagnostic performance in predicting ICH patients with poor outcomes (sensitivity = 64.3%; specificity = 80.8%; area under curve (AUC) = 0.742, 95% CI: 0.567-0.916).

## 4. Discussion

In this study, we used integrative analyses of transcriptomics and proteomics to explore molecular mechanisms, underlying the development of ICH-induced brain injury. Using the collagenase model of ICH on mice, we determine the following: (1) immune inflammatory response and coagulation cascade activation were deeply involved in the acute stage of ICH; (2) an ICH-related rCCA network involving two highly connective clusters was presented by integrative multiomics analyses; and (3) Serpina3n, Itgb2, and Ctss were identified as hub molecules at both omics levels, and Serpina3n could be a promising biomarker in the diagnosis and prognosis of ICH.

Based on bioinformatics analysis of transcriptomic and proteomics profiles, our results showed that cytokine-cytokine receptor interaction, complement, and coagulation cascade pathways were significantly enriched at both mRNA and protein levels in ICH compared with the sham operated animals, which demonstrated an activation of numerous inflammatory cascades occurred in the perihematomal area of brains post-ICH. The cascades include the activation of microglia, astrocytes, and the infiltration of leukocytes [28]. Activated immune cells release a mass of proinflammatory cytokines and chemokines into the brain. These molecules aggravate the initial inflammatory response, which lasts over days or even weeks and is able to affect the subsequent brain repair processes [28]. Besides, the hematoma around the ruptured blood vessel could trigger the coagulation cascade and release a large amount of thrombin, which eventually leads to perihematomal edema and neurological deficit [12]. Strikingly, our results showed that pyroptosis, a newly discovered inflammatory programmed cell death pathway, might be involved in the occurrence and progression of ICH. Previous studies reported that inflammasome-mediated pyroptosis was an important event that led to neuronal and microglial cell death and consequently to the release of proinflammatory cytokines and neuroinflammation [29–31]. The results mentioned above suggested that immune and agglutination-related processes played important roles in ICH pathology.

Given the gap in the depth and breadth between proteome and transcriptome, separated omics might overlook the crosstalk between different molecular entities and could miss biologically relevant information [22]. In this context, we constructed an integrated rCCA network of ICH through a multiomics approach of combing DEG and DEP datasets, which revealed pathological changes involved in ICH from a more comprehensive perspective. We constructed the rCCA network of DEGs and DEPs, which identified important modules related to ICH pathology. In general, we found some well-known pathological changes after ICH, such as wounding response and acute inflammatory response, which had been comprehensively described in previous researches. That fact supports the reliability of our results and the robustness of methodological approaches. Particularly, we identified a cluster of interconnected molecules, which was greatly involved in sphingolipid catabolic process, such as SMPDL3B. Sphingolipids are not only important cell membrane components but also key regulator molecules of neural signal transduction and cell differentiation. A recent study showed that catabolic disorder of sphingomyelin could decrease cell membrane integrity and hinder neural differentiation, mainly due to the reduction of SMPDL3B [32]. Sphingosylphosphorylcholine (SPC), a kind of sphingolipids, could mediate vascular inflammation by increasing the release of MCP-1 and might contribute to vasospasm events after SAH [33]. Besides, cleavage of membrane sphingomyelin by sphingomyelinase generated the second messenger ceramide (Cer), which is involved in an injury-responsive signaling pathway and act as a trigger of mediated neuronal apoptosis and IL-6 expression after ischemic stroke [34, 35]. Thus, we hypothesized that accumulation of sphingolipids might impair neural differentiation and cause excessive Cer production and eventually contribute to neuroinflammation and induce cell apoptosis in ICH.

Integromics-based candidates could be biologically relevant regardless of whether the changes at each single omics level are large or small [22]. In our study, we found three potential candidates (Serpina3n, Itgb2, and Ctss) with high degree scores in the PPI network or high weight in the rCCA network. We also successfully validate that these candidates were significantly upregulated at both mRNA and protein levels following ICH.

Itgb2 (CD18), a cell-surface glycoproteins, belongs the integrin family and acts as a coordinator of extra- and intracellular signaling. It has been reported that itgb2 could interact with intercellular adhesion molecule-1 (ICAM-1) to promote firm adhesion of neutrophils to the endothelium in acute inflammation [36]. Moreover, itgb2 deficiency could also exert a neuroprotective effect via decreasing numbers of infiltrating leukocytes, reducing the release of inflammatory cytokines and chemokines [37–39]. We have observed a significant increase of itgb2 in both gene expression and protein abundance in ICH brain tissues, indicating that itgb2 might play a role in regulating signal transduction of immune cells and mediating neuroinflammation in the acute phase of ICH. However, the signal downstream involved in itgb2 after ICH remained obscure and needed to be further explored.

Ctss is a lysosomal protease with highly specific proteolytic activity [40]. Its activation might result in the initiation of cancer, abdominal aortic aneurysm, and atherosclerosis through regulating tumor-promoting immune microenvironment and extracellular matrix degradation [41–44]. As a proinflammatory mediator, Ctss is able to secrete from activated microglia into the extracellular space and subsequently contribute to the impairment of the BBB, and Ctss inhibitor is reported to ameliorate BBB injury and neurological dysfunction in ischemic stroke [45]. Given the fact that cerebral inflammation and BBB disruption are major pathophysiological events in ICH, it is worth to explore the underlying mechanism of Ctss following hemorrhagic brain injury in future studies. Besides, the inhibition of Ctss to restore BBB function and neurological deficits might be an effective therapeutic intervention after ICH.

Murine Serpina3n, the homologue of human SERPINA3 (a1-antichymotrypsin (ACT)), is a member of the superfamily of serpins, which is highly expressed in the brain, testis, lungs, thymus, and spleen [46, 47]. Serpina3n is reported to predominantly targets multiple types of immune cells and inhibits the release of serine protease, such as cathepsin G (CatG), leukocyte elastase (LE), granzyme B (GrB), and matrix metalloproteinase 9 (MMP9), exerting the function in the maintenance of immune balance accordingly [48–50]. In our study, we observed a rapid upregulation of Serpina3n mRNA and protein levels in brain tissues of ICH mice at the acute phase, suggesting the contribution of Serpina3n to participate in regulating early immune response following the insult. Indeed, it has been previously reported that overexpressed Serpina3n could be induced by glia cells in the central nervous [51]. Another research on ischemic stroke has also demonstrated that Serpina3n could be a potent marker in place of conventional biomarker GFAP for its specific identification of activated astrocytes [52]. However, no relationship has been identified between Serpina3n and ICH-induced injury yet. Accordingly, there is a need to dig into the exact mechanism by which Serpina3n can produce a benefit or harmful effect on neurological function following ICH onset.

Based on preliminary findings of the studies mentioned above and Serpina3n results of our study, we subsequently measured its expression levels in the plasma of ICH patients to decipher whether the Serpina3n/SERPINA3 could achieve a translation from animal discovery to human research. Of interest, we found that ICH patients exhibited elevated plasma Serpina3 levels compared with the controls, and the increased level of SERPINA3N in plasma of ICH patients was highly associated with poorer outcome (mRS score 4-6). It has been previously reported that SERPINA3 was identified as a potential diagnostic biomarker in other neurological disorders, such as multiple sclerosis and Alzheimer's disease [53, 54]. In fact, after ICH onset, peripheral lymphocytes could be soon activated and subsequently infiltrated into the brain parenchymalocal, accompanied by the release of massive cytokines, chemokines, and proteases [55], which might prompt the rapid and persistent secretion of Serpina3n to regulate the balance of the immune system. In this context, circulating Serpina3n/SERPINA3 levels might be a promising monitoring marker of prognosis after ICH.

Several limitations of the present study need to be borne in mind. First, although integrative approaches are implemented to gain a deep insight into ICH pathology underlying multiple molecular levels, there is a need for further exploration of methods that combine omics data with ICH phenotypic data. Second, due to the acquirement limitation of clinical samples, we were not able to exhibit cerebrospinal fluid (CSF) SERPINA3 concentrations during ICH progression. Lastly, the study cohort of ICH patients and the controls was relatively small, a larger-scale enrollment should be taken to further investigate the diagnostic efficacy of candidate SERPINA3.

## 5. Conclusions

Using integrative multiomics approaches, we effectively identified hub genes and proteins and revealed an ICH-related molecule network in brain of mice. Moreover, we demonstrated that Serpina3n/SERPINA3 is a potential biomarker, in which expression is associated with the diagnosis and prognosis for ICH patients.

## Figures and Tables

**Table 1:** GO and KEGG enrichment analysis of DEGs following ICH.

Ontology	ID	Description	GeneRatio	BgRatio	*p* value	*p*.adjust	*q* value
BP	GO:0050900	Leukocyte migration	92/1311	340/23210	8.65*e* − 38	4.39*e* − 34	3.28*e* − 34
BP	GO:0002274	Myeloid leukocyte activation	72/1311	215/23210	1.74*e* − 36	4.41*e* − 33	3.30*e* − 33
BP	GO:0001819	Positive regulation of cytokine production	99/1311	449/23210	1.78*e* − 32	3.00*e* − 29	2.25*e* − 29
BP	GO:0031349	Positive regulation of defense response	84/1311	370/23210	1.24*e* − 28	1.57*e* − 25	1.18*e* − 25
BP	GO:0007159	Leukocyte cell-cell adhesion	77/1311	319/23210	3.46*e* − 28	3.52*e* − 25	2.63*e* − 25
CC	GO:0062023	Collagen-containing extracellular matrix	59/1322	359/23436	1.17*e* − 13	4.20*e* − 11	3.56*e* − 11
CC	GO:0031012	Extracellular matrix	70/1322	475/23436	1.57*e* − 13	4.20*e* − 11	3.56*e* − 11
CC	GO:0000779	Condensed chromosome, centromeric region	20/1322	51/23436	1.34*e* − 12	2.16*e* − 10	1.83*e* − 10
CC	GO:0000775	Chromosome, centromeric region	39/1322	190/23436	1.61*e* − 12	2.16*e* − 10	1.83*e* − 10
CC	GO:0098687	Chromosomal region	49/1322	299/23436	1.58*e* − 11	1.70*e* − 09	1.44*e* − 09
MF	GO:0008009	Chemokine activity	20/1278	44/22710	4.23*e* − 14	1.87*e* − 11	1.57*e* − 11
MF	GO:0004896	Cytokine receptor activity	29/1278	97/22710	4.53*e* − 14	1.87*e* − 11	1.57*e* − 11
MF	GO:0019955	Cytokine binding	32/1278	129/22710	6.89*e* − 13	1.89*e* − 10	1.59*e* − 10
MF	GO:0005125	Cytokine activity	41/1278	215/22710	4.93*e* − 12	1.01*e* − 09	8.54*e* − 10
MF	GO:0042379	Chemokine receptor binding	20/1278	67/22710	4.01*e* − 10	6.61*e* − 08	5.57*e* − 08
KEGG	mmu04060	Cytokine-cytokine receptor interaction	70/582	302/8910	1.11*e* − 21	3.15*e* − 19	2.46*e* − 19
KEGG	mmu04061	Viral protein interaction with cytokine and cytokine receptor	35/582	103/8910	7.27*e* − 17	1.03*e* − 14	8.03*e* − 15
KEGG	mmu04640	Hematopoietic cell lineage	30/582	96/8910	1.58*e* − 13	1.49*e* − 11	1.16*e* − 11
KEGG	mmu05152	Tuberculosis	41/582	180/8910	7.97*e* − 13	5.64*e* − 11	4.41*e* − 11
KEGG	mmu05323	Rheumatoid arthritis	26/582	87/8910	2.12*e* − 11	1.20*e* − 09	9.37*e* − 10

**Table 2:** GO and KEGG enrichment analysis of DEPs following ICH.

Ontology	ID	Description	GeneRatio	BgRatio	*p* value	*p*.adjust	*q* value
BP	GO:0007596	Blood coagulation	19/333	156/23210	1.18*e* − 12	2.12*e* − 09	1.50*e* − 09
BP	GO:0007599	Hemostasis	19/333	158/23210	1.49*e* − 12	2.12*e* − 09	1.50*e* − 09
BP	GO:0050817	Coagulation	19/333	159/23210	1.67*e* − 12	2.12*e* − 09	1.50*e* − 09
BP	GO:0042060	Wound healing	27/333	360/23210	2.87*e* − 12	2.74*e* − 09	1.94*e* − 09
BP	GO:0009611	Response to wounding	31/333	495/23210	7.37*e* − 12	5.63*e* − 09	3.98*e* − 09
CC	GO:0062023	Collagen-containing extracellular matrix	29/339	359/23436	8.19*e* − 14	3.58*e* − 11	2.56*e* − 11
CC	GO:0031012	Extracellular matrix	32/339	475/23436	5.96*e* − 13	1.30*e* − 10	9.31*e* − 11
CC	GO:0015629	Actin cytoskeleton	23/339	490/23436	8.83*e* − 07	1.29*e* − 04	9.20*e* − 05
CC	GO:0005764	Lysosome	21/339	431/23436	1.48*e* − 06	1.34*e* − 04	9.57*e* − 05
CC	GO:0000323	Lytic vacuole	21/339	432/23436	1.53*e* − 06	1.34*e* − 04	9.57*e* − 05
MF	GO:0003779	Actin binding	25/327	428/22710	3.77*e* − 09	2.39*e* − 06	1.96*e* − 06
MF	GO:0004857	Enzyme inhibitor activity	22/327	384/22710	4.70*e* − 08	1.44*e* − 05	1.18*e* − 05
MF	GO:0061135	Endopeptidase regulator activity	16/327	209/22710	6.80*e* − 08	1.44*e* − 05	1.18*e* − 05
MF	GO:0061134	Peptidase regulator activity	17/327	244/22710	1.04*e* − 07	1.65*e* − 05	1.35*e* − 05
MF	GO:0004867	Serine-type endopeptidase inhibitor activity	11/327	118/22710	1.13*e* − 06	1.44*e* − 04	1.18*e* − 04
KEGG	mmu04610	Complement and coagulation cascades	15/169	93/8910	1.72*e* − 10	4.38*e* − 08	4.12*e* − 08
KEGG	mmu04611	Platelet activation	10/169	124/8910	1.17*e* − 04	0.015	0.014
KEGG	mmu04142	Lysosome	10/169	131/8910	1.85*e* − 04	0.016	0.015
KEGG	mmu04612	Antigen processing and presentation	8/169	91/8910	3.17*e* − 04	0.020	0.019
KEGG	mmu05133	Pertussis	7/169	77/8910	6.15*e* − 04	0.031	0.029

**Table 3:** Biological processes involved in cluster 1 and cluster 2.

	GO term (biological process)	*p* value	Corrected *p* value	Cluster frequency	Genes
Network 1	Sphingolipid catabolic process	6.99*E* − 05	1.57*E* − 02	2/28, 7.1%	GALC, SMPDL3B
	Membrane lipid catabolic process	8.15*E* − 05	1.57*E* − 02	2/28, 7.1%	GALC, SMPDL3B
	Cerebellar cortex morphogenesis	1.37*E* − 04	1.57*E* − 02	2/28, 7.1%	AGTPBP1, SERPINE2
	Neurotransmitter metabolic process	1.69*E* − 04	1.57*E* − 02	2/28, 7.1%	AGTPBP1, TH
	Cerebellum morphogenesis	2.25*E* − 04	1.57*E* − 02	2/28, 7.1%	AGTPBP1, SERPINE2
Network 2	Response to wounding	4.76*E* − 08	6.15*E* − 05	6/20, 30.0%	APOH, SERPING1, SERPINA3N, CFP, CXCL3, AQP1
	Response to stress	6.77*E* − 08	1.81*E* − 03	7/20, 35.0%	ANXA1, APOH, SERPING1, SERPINA3N, CFP, CXCL3, AQP1
	Inflammatory response	9.49*E* − 08	1.81*E* − 03	4/20, 20.0%	SERPING1, SERPINA3N, CFP, CXCL3
	Acute inflammatory response	2.04*E* − 07	1.81*E* − 03	3/20, 15.0%	SERPING1, SERPINA3N, CFP
	Response to stimulus	2.04*E* − 07	1.81*E* − 03	9/20, 45.0%	ANXA1, ARG1, APOH, SERPING1, SERPINA3N, CFP, CXCL3, XDH, AQP1

GO: gene ontology.

## Data Availability

The data used to support the findings of this study are available from the corresponding authors upon request.

## Abbreviations

ICH: Intracerebral hemorrhage
BBB: Blood-brain barrier
ECCMU: Ethical Committee of Chongqing Medical University
LC-MS/MS: Liquid chromatography-mass spectrometry/mass spectrometry
qRT-PCR: Quantitative RT-PCR
H&E: Hematoxylin eosin
PBS: Phosphate-buffered saline
DEGs: Differentially expressed genes
FDR: False discovery rate
DEPs: Differentially expressed proteins
GO: Gene ontology
KEGG: Kyoto Encyclopedia of Genes and Genomes
GSEA: Gene Set Enrichment Analysis
PPI: Protein-protein interaction network
rCCA: Regularized Canonical Correlation Analysis
BiNGO: Biological Networks Gene Ontology
BCA: Bicinchoninic acid
ELISA: Enzyme-linked immunosorbent assay
mRS: Modified Rankin Scale
PCA: Principal component analysis
BP: Biological process
CC: Cellular component
MF: Molecular function
ES: Enrichment scores
GCS: Glasgow Coma Scale
NIHSS: National Institute of Health Stroke Scale
IVH: Intraventricular hemorrhage
CT: Computed tomography
ROC: Receiver operating characteristic
AUC: Area under curve
SPC: Sphingosylphosphorylcholine
Cer: Ceramide
ICAM-1: Intercellular adhesion molecule-1
ACT: a1-Antichymotrypsin
CatG: Cathepsin G
LE: Leukocyte elastase
Grb: Granzyme B
MMP9: Matrix metalloproteinase 9
CSF: Cerebrospinal fluid.

## Supplementary Materials

Supplementary Materials Table S1: primer sets for quantitative real-time PCR. Table S2: comparison of the demographic and clinical characteristics between patients with ICH and health control group. Table S3: comparison of the demographic and clinical characteristics in ICH patients according to 90-day outcome. Figure S1: the correlation analysis between ten hub gene expression and behavior tests.
