# Supplementary material for: Integrated Multiomics Analysis Identifies a Novel Biomarker Associated with Prognosis in Intracerebral Hemorrhage
Source: Oxid Med Cell Longev. 2021 Dec 14;2021:2510847. doi: 10.1155/2021/2510847 (PMC8691985; doi:10.1155/2021/2510847)
Supplement: Supplementary Materials — Table S1: primer sets for quantitative real-time PCR. Table S2: comparison of the demographic and clinical characteristics between patients with ICH and health control group. Table S3: comparison of the demographic and clinical characteristics in ICH patients according to 90-day outcome. Figure S1: the correlation analysis between ten hub gene expression and behavior tests. [file 2510847.f1.doc]

Supplementary Table S1: Primer sets for quantitative real-time PCR.

| Gene | Forward | Reverse |
| --- | --- | --- |
|
| Lyz2 | AGGTCTATGAACGTTGTGAGTT | ACCAGTATCGGCTATTGATCTG |
| C3 | TCCAACAAGAACACCCTCA | GGCTGGATAAGTCCCACA |
| Serpina3n | GTCTCTGAGGAAATGGAGGAAA | GGAGAAGACTTCCTTGATACCC |
| Cfp | GTATCAAAACGGATTGTGACGT | TAACACATGTCACAGTGTCTGA |
| Ppbp | CTCAGACCTACATCGTCCTGC | GTGGCTATCACTTCCACATCAG |
| Haptoglobin | GCTATGTGGAGCACTTGGTTC | CACCCATTGCTTCTCGTCGTT |
| Serping1 | TAGAGCCTTCTCAGATCCCGA | ACTCGTTGGCTACTTTACCCA |
| Ctss | CCATTGGGATCTCTGGAAGAAAA | TCATGCCCACTTGGTAGGTAT |
| Itgb2 | AGGAGCATCGCTAATCCTGAG | CCTGGTCGCAAGTAAAGTGTC |
| Anxa2 | ATGTCTACTGTCCACGAAATCC | CTTGACTGCTGTCTCAATGTTC |
| β-actin | GCGGGCGACGATGCT | TGCCAGATCTTCTCCATGTCG |

Supplementary Table S2: Comparison of the demographic and clinical characteristics between patients with ICH and Health control group.

|  | ICH  (n=40 , 51.3%) | Control  (n=38 , 48.7%) | *p*-value |
| --- | --- | --- | --- |
|
| Demographic |  |  |  |
| Mean age, y (SD) | 64.6 (7.8) | 64.0 (10.1) | 0.778 |
| Sex, male, n (%) | 25 (62.5) | 24 (63.2) | 0.952 |
| Clinical characteristics |  |  |  |
| Diabetes mellitus, n (%) | 7 (17.5) | 5 (13.2) | 0.595 |
| History of hypertension, n (%) | 28 (70.0) | 8( 21.1) | <0.001 |
| Serpina3n, ng/mL (SD) | 13.3 (2.5) | 11.2 (3.8) | 0.015 |

ICH: intracerebral hemorrhage, SD: standard deviation,

SUPPLEMENTARY TABLE S3: Comparison of the demographic and clinical characteristics in ICH patients according to 90-day outcome.

|  | Poor outcome  (n=14, 35%) | Good outcome  (n=26, 65%) | *p*-value |
| --- | --- | --- | --- |
|
| Demographic |  |  |  |
| Mean age, y (SD) | 64.1 (10.5) | 64.0 (10.0) | 0.776 |
| Sex, male, n (%) | 7 (50%) | 18 (69.2%) | 0.231 |
| Clinical characteristics |  |  |  |
| Alcohol consumption, n (%) | 3 (21.4) | 11 (42.3) | 0.331 |
| Smoking, n (%) | 4 (28.6) | 12 (46.2) | 0.279 |
| Diabetes mellitus, n (%) | 2 (14.3) | 5 (19.2) | 0.507 |
| History of hypertension, n (%) | 10 (71.4) | 18 (69.2) | 1.000 |
| Admission SBP, mmHg (SD) | 185.4 (41.3) | 176.1 (20.8) | 0.081 |
| Admission DBP, mmHg (SD) | 99.0 (26.1) | 102.1 (14.4) | 0.932 |
| Admission GCS score, median (IQR) | 10 [7.3-12.8] | 15 [13.3-15.0] | <0.001 |
| Admission NIHSS score, median (IQR) | 19.5 [11.3-25.0] | 8 [4-13.8] | <0.001 |
| Imaging features |  |  |  |
| Baseline ICH volume, mL(IQR) | 17.4 [7.3-33.8] | 8.0 [3.1-15.8] | 0.029 |
| Presence of IVH on initial CT, n (%) | 8 (57.1) | 2 (7.7) | 0.002 |
| ICH Locations |  |  |  |
| Lobar hemorrhage, n (%) | 0 (0) | 1 (3.8) | 1.000 |
| Deep hemorrhage, n (%) | 11 (78.6) | 20 (73.1) | 1.000 |
| Infratentorial hemorrhage, n (%) | 3 (4.6) | 5 (23.1) | 0.847 |
| Plasma Serpina3n levels (ng/mL) | 14.6 (2.4) | 12.7 (2.4) | 0.022 |

ICH: intracerebral hemorrhage, GCS: Glasgow Coma Scale, IVH: intraventricular hemorrhage, IQR: inter-quartile range, SD: standard deviation, mRS: modified Rankin scale, SBP: systolic blood pressure, DBP: diastolic blood pressure.


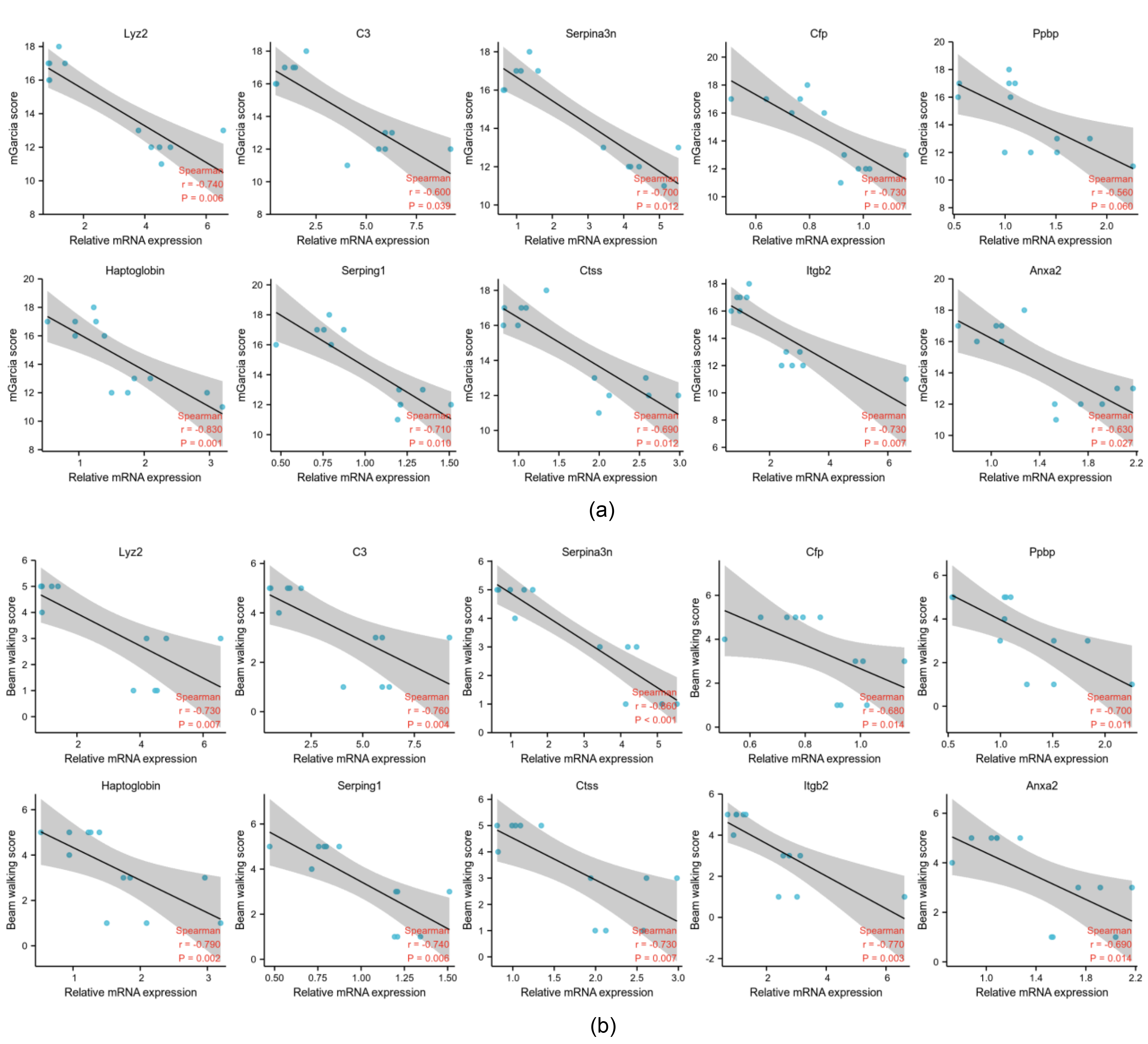


SUPPLEMENTARY FIGURE 1: The correlation analysis between ten hub genes expression and behavior tests. (a) Spearman correlation analysis results showing the correlation between the mRNA expression levels of hub genes and the scores of Garcia test. (b) Spearman correlation analysis results showing the correlation between the mRNA expression levels of hub genes and the scores of Beam walking test. *, *p* < 0.05; **, *p* < 0.01; ***, *p* < 0.001.
